# Mature Ovarian Teratoma: Atypical Imaging

**DOI:** 10.1155/2020/1352961

**Published:** 2020-02-18

**Authors:** S. Wakrim, M. EL Jdid

**Affiliations:** ^1^Radiology Department, Faculty of Medicine and Pharmacy, Ibn Zohr University, Agadir, Morocco; ^2^Pediatric Surgery Department, Regional Hospital Center Hassan 2, Agadir, Morocco

## Abstract

The incidence of a mature ovarian teratoma ranged from 20% to 30% of pediatric ovarian tumors (Sabaa et al., 2009), which is composed of well-differentiated tissues that derive from all three germ cell layers (ectoderm, mesoderm, and endoderm); it is one of the most common benign ovarian neoplasms. In this case report, we discuss a 9-year-old female patient who presented with abdominal pain and distended abdomen, for which she had an abdominal ultrasound and magnetic resonance imaging. The histopathological exam, after a laparotomy, showed a mature ovarian teratoma.

## 1. Introduction

Germ cell tumors are the most common ovarian neoplasms in childhood and adolescence, and of these, the teratomas, whether mature or immature, are the most frequently found [1]. Teratomas are often composed of multiple embryologic layers, which arise from multipotent cells, and are divided into both mature and immature forms [2].

A mature teratoma (MT), which is composed of well-differentiated tissues that derive from all three germ cell layers (ectoderm, mesoderm, and endoderm), is one of the most common benign ovarian neoplasms [3, 4].

In most cases, they are easily diagnosed on imaging studies because of their characteristic intratumoral fat component. Although typical imaging findings of mature cystic teratomas are well known to radiologists, various atypical imaging features can be particularly misleading. This article illustrates atypical imaging manifestations of mature ovarian cystic teratomas through a clinical case and literature review.

## 2. Case Description

We present the case of a 9-year-old female patient who presented with diffuse abdominal pain, distended abdomen, and rapid increase in abdominal volume.

**Figure 1:**
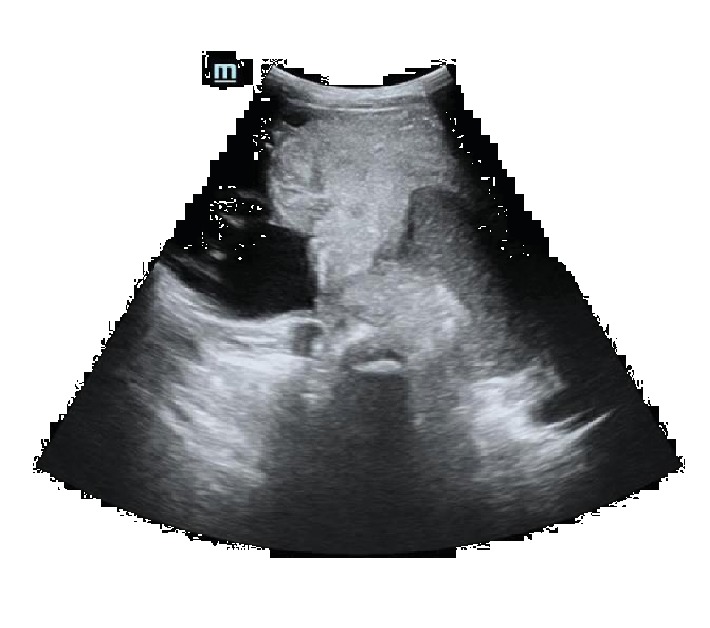
Abdominal ultrasound demonstrates an abdominopelvic mass with different compositions.

An abdominal ultrasound diagnosed an abdominopelvic mass with different compositions (Figure 1).

**Figure 2:**
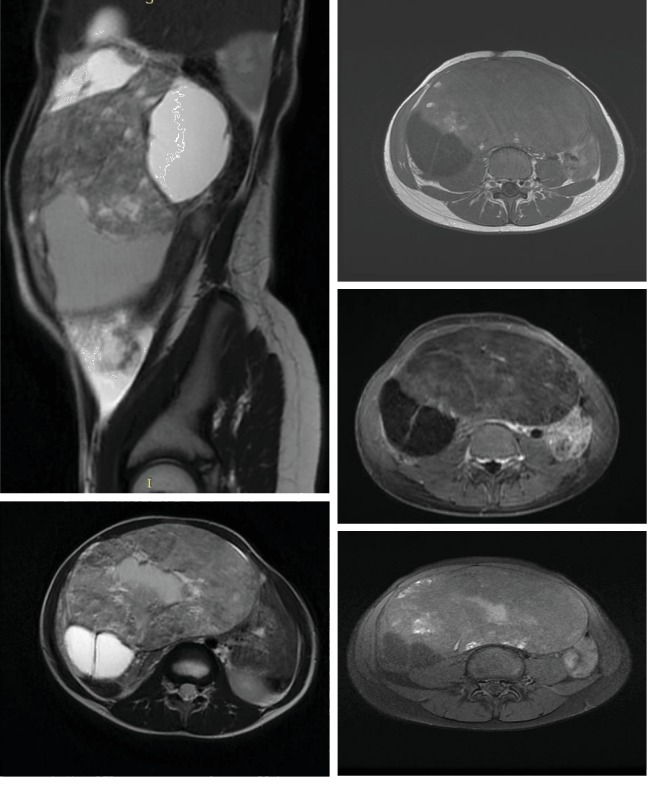
Axial T1-weighted, axial contrast-enhanced fat-suppressed T1-weighted, axial T2-weighted, sagittal T2-weighted, and contrast-enhanced T1-weighted magnetic resonance (MR) images demonstrate a solid cystic mass of approximately 126 mm in diameter in the abdominopelvic cavity. The cystic structures are of different signal, and the solid mass is raised discretely after injection of contrast medium, probably originating from the left ovary.

Magnetic resonance imaging was performed using 1.5 T MRI with sagittal, transverse T2-weighted, and transverse T1-weighted turbo spin-echo and transverse fat-suppressed T1-weighted turbo spin-echo images. After rapid bolus intravenous injection of gadolinium, transverse contrast-enhanced fat-suppressed T1-weighted turbo spin-echo images and sagittal contrast-enhanced T1-weighted turbo spin-echo images were obtained (Figure 2).

The images demonstrate a solid cystic mass of approximately 126 mm in diameter in the abdominopelvic cavity.

The cystic structures are of different signal, and the solid mass is raised discretely after injection of contrast medium, probably originating from the left ovary. As a result, the diagnosis proposed is an immature ovarian teratoma.

**Figure 3:**
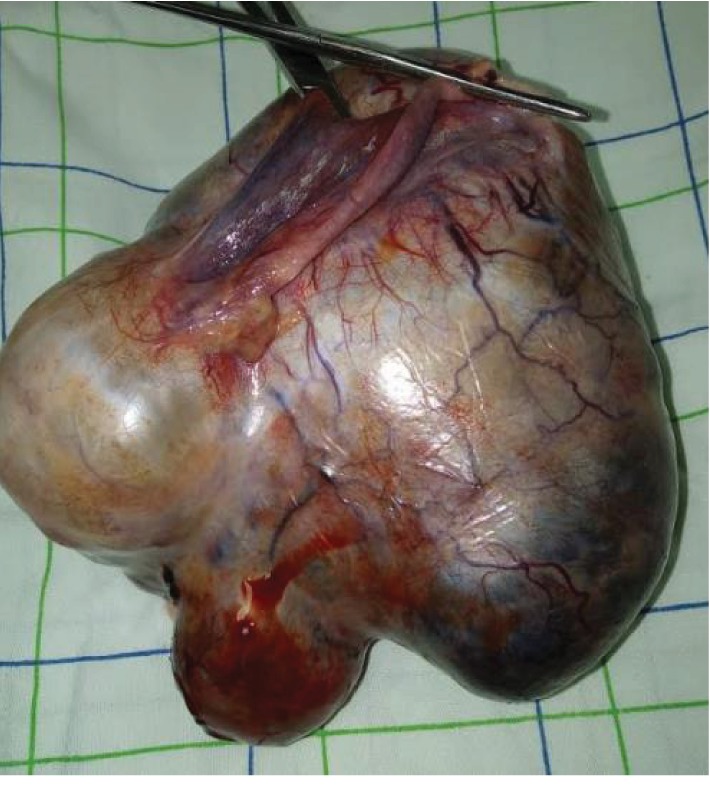
The surgical specimen consists of the left ovary. The cyst wall is smooth and glistening.

A laparotomy was performed, and the patient underwent left oophorectomy. The histopathological exam pointed out a mature ovarian teratoma (Figure 3).

## 3. Discussion

The incidence of a mature ovarian teratoma ranged from 20% to 30% of ovarian tumors in children [5]. Bilaterality is between 8 and 15%. They consist of well-differentiated tissues from at least 2 of 3 layers of stem cells. Ectodermal and mesodermal tissues are found in 100% and 90% of cases, respectively, and endodermal tissues in the majority of cases [6].

Macroscopically, the mature teratoma is a cystic tumor in 88% of cases, rarely solid. Its wall is covered with a squamous epithelium derived from the ectoderm and limited outside by the ovarian stroma packed around the cyst [7]. Its content is liquid, most often sebaceous type and much more rarely serous type. You can also find hair in the cyst. Quite frequently, a solid nodule is attached to the inner side of the cystic wall, called the Rokitansky nodule or protuberance. It is in this protuberance that the derivatives of 3 layers of stem cells are found: nerve tissue, dander, adipose tissue, gastrointestinal mucosa, etc. The visible hairs within the cyst are derived from this nodule.

A minor percentage of mature cystic teratomas have only a small amount of fat or no visible fat on imaging studies.

A mature teratoma is a benign tumor, whereas the immature type, although also benign, has a more aggressive course, with a propensity to recurrence [8].

It is important to remember that the diagnosis of a mature teratoma (MT) may be overlooked in the small number of tumors that do not contain fat and that the differentiation from other epithelial neoplasms is difficult [8]. Sometimes it is possible to detect small amounts of fat in the wall: the cystic wall must be carefully examined to do the correct preoperative diagnosis. To show a small amount of fat on MR images, a gradient-echo technique with both in-phase and opposed-phase imaging is very useful. This technique is probably superior to the use of fat saturation method [9–11]. So, in those patients showing the absence of fat in the cyst, the identification of fat in the cyst wall, by using gradient-echo sequences, is important to obtain the correct diagnosis.

In some cases, MTs show atypical imaging manifestation. It is possible to distinguish the atypical manifestation caused by tumor components: MTs without fat in the cystic cavity, MTs with a pure fatty component in the cyst, and combination and collision tumors [12, 13]. A minor percentage of MTs have only a small amount of/or no visible fat on imaging studies, but rarely MTs have a pure fat component on imaging without showing any other components: these MTs may mimic other rare lipid-containing tumors (benign pelvic lipoma, liposarcoma).

## 4. Conclusion

Imaging findings of mature cystic teratomas can be atypical depending on the tumor components. Understanding the atypical imaging manifestations of mature cystic teratomas permits a more specific and accurate diagnosis.
